# BioProEV: A Bioinformatics Pipeline for Biologically‐Relevant Handling of Missing Values in the Analysis of Extracellular Vesicles by Mass Spectrometry

**DOI:** 10.1002/jex2.70150

**Published:** 2026-05-15

**Authors:** Pâmella Miranda, Jose G. Marchan‐Alvarez, Annemarijn Offens, Ruihan Zhou, Mathieu Y. Brunet, Loes Teeuwen, Maria Eldh, Susanne Gabrielsson, Phillip T. Newton

**Affiliations:** ^1^ Department of Women's and Children's Health Karolinska Institutet Stockholm Sweden; ^2^ Astrid Lindgren Children's Hospital Stockholm Sweden; ^3^ Division of Immunology and Allergy Department of Medicine (Solna) Karolinska Institutet Stockholm Sweden; ^4^ Clinical Immunology and Transfusion Medicine Karolinska University Hospital Stockholm Sweden

**Keywords:** Bovine milk, extracellular vesicles, fetal bovine serum (FBS), missing values, proteomics

## Abstract

While proteomics is being increasingly applied to investigate extracellular vesicles (EVs), there remains no consensus on how to address the inevitable missing values within proteomics datasets. Here, we devised a three‐step approach that prioritized retaining biologically‐relevant information in EV samples using a dataset containing two populations of EVs analysed by liquid chromatography electrospray ionization tandem mass spectrometry (LC‐ESI‐MS/MS). Firstly, to avoid overfitting, we excluded proteins in which more than half of the values were missing. Next, we statistically tested for low protein abundance in a single population: when the number of potential “missing not at random” (MNAR) values was significantly enriched, these values were replaced with the lowest possible value of “1”. Finally, all remaining missing values were then imputed using the Random Forest machine learning algorithm. Our final dataset included 49.9 % of proteins that originally contained at least one missing value, from which 84.7 % were listed in the ExoCarta database, significantly more than would be expected by chance, strongly indicating biologically relevant imputation. To enable other EV researchers to analyse proteomics data in a robust, easy‐to‐use and peer‐reviewed manner, we provide BioProEV, a bioinformatics pipeline to impute missing values with biological relevance in EV datasets.

## 1 Introduction

Extracellular vesicles (EVs) are small membrane‐bound particles, released by virtually all cells into the extracellular space, that are increasingly recognized as important mediators of intercellular communication since they contain biologically active molecules including nucleic acids, proteins and lipids. Among these, proteins, both internal and surface‐related, are integral components of EVs that can determine their properties and functions, and therefore, important information can be obtained by analysing the protein compositions of EV preparations (Iraci et al. 2016; Yáñez‐Mó et al., 2015). For example, some proteins are typically enriched in EVs independently of their cellular origin; these proteins, such as the tetraspanins (e.g. CD63, CD81 and CD9), and cytosolic proteins (e.g. Annexins and PDCD6IP [also known as ALIX]), are often used to ascertain whether or not isolated nanoparticles can be identified as EVs (Welsh et al. 2024). Additionally, certain proteins present in EVs reflecting their cell of origin, such that EVs obtained from biological fluids like blood and urine, can be used diagnostically or prognostically to identify pathological changes (Kumar et al. 2024). For example, circulating EVs enriched in cysteine‐rich protein 61 (Cyr61), human ether‐a‐go‐go‐related gene 1 (hERG1) and heat shock protein 47 (Hsp47) have predictive potential for cardiovascular diseases (W. Li et al. 2021; Osorio et al. 2024), while other proteins such as alpha 1‐antitrypsin, Histone H2B type 1‐K (H2B1K) and Tumour‐associated calcium signal transducer 2 (TACSTD2) in urinary EVs have been identified as diagnostic and prognostic biomarkers for bladder cancers (Chen et al. 2012; Lin et al. 2016). Furthermore, other proteins can confer functional properties to EVs under both physiological and pathological conditions, such as the modulation of immune function (Kalluri 2024) or the regulation of mineralization of extracellular matrix during skeletal growth (Cui et al. 2016), as well as stimulating liver metastases (H. Zhang et al. 2017) or promoting atherosclerotic plaque reactivity (Oggero et al. 2021). Hence, proteomics is becoming an increasingly important tool for EV researchers to explore the roles of EVs in both physiological and pathological settings, and robust approaches to analyse proteomics data are needed to allow meaningful interpretation and reproducibility.

Missing values are absences of data within a dataset in which individual samples lack a numerical score for a certain protein, and are inevitable in proteomics datasets (Karpievitch et al. 2012; Karpievitch et al. 2009; Webb‐Robertson et al. 2015). While the number of missing values in proteomics data varies, it is usually greater than 50 % of the total data, with at least 50 % of the given proteins (variables) likely to have at least one missing value (Albrecht et al. 2010; Karpievitch et al. 2009; Lazar et al. 2016; Liu and Dongre 2021). This absence in the data can greatly affect the downstream analyses, reducing the power of statistical tests and leading to bias in the interpretation of results (Albrecht et al. 2010; Jin et al., 2021; Zhou et al. 2018). Detection and quantification of EV proteins is more challenging than cell‐derived proteins, as no available method can purify EVs completely and different purification technologies could result in different proteomic analysis results. Preparations from biological fluids with lower EV concentrations are likely to contain relatively more contaminating non‐EV‐related proteins than those with higher EV concentrations, leading to an increased chance of missing values across samples. Furthermore, normalization of input material based on original bodily fluid volume, protein concentration or particle numbers all have their challenges and limitations. Despite this, missing value handling is frequently not addressed in methodological descriptions within the EV literature, compromising the reliability and reproducibility of published studies (Karimi et al. 2018; Kowal et al. 2016; Kreimer et al. 2015; Singh et al. 2025).

Missing values are caused by biological and analytical factors, including the absence of a protein from a sample, protein abundance below the instrument's detection limit, sample loss in preparation, miscleavage of peptides during the digestion process, and instrument calibration (Albrecht et al. 2010; Jin et al., 2021; Karpievitch et al. 2012; Zhou et al. 2018). The missing values can be classified by three missingness mechanisms: whereas missing at random (MAR, which depends only on the observed data and is related to sample preparation or instrument settings) and missing completely at random (MCAR, which does not depend on either observed or missing data) both appear randomly in the dataset, missing not at random (MNAR, which depends only on the missing data) is caused by relatively low protein abundance for biological reasons (Little and Rubin, 2019; RUBIN 1976). In proteomics data, missing values usually represent a mixture of MNAR (protein abundance‐dependence) and MCAR/MAR (protein abundance‐independence) (Albrecht et al. 2010; Jin et al., 2021; Karpievitch et al. 2012; Webb‐Robertson et al. 2015).

Since there is no rule of thumb for handling missing data (Jin et al., 2021; Webb‐Robertson et al. 2015), we developed a novel approach to investigate proteomics data obtained from two populations of EVs. Thus, we aimed to devise a method to handle missing values in order to maintain meaningful biological differences in the EV data while minimizing the influence of imputation methods to introduce spurious results. We combined these steps into a bioinformatics pipeline, named BioProEV, which we applied to two EV populations, fetal bovine serum (FBS) and bovine milk EVs (each analysed in triplicate), obtained from liquid chromatography electrospray ionization tandem mass spectrometry (LC‐ESI‐MS/MS).

## 2 Methods

### 2.1 Standardization of Experimental Parameters of EV Studies

To contribute to transparency and reproducibility in the EV field, all experimental details have been submitted to the EV‐TRACK knowledgebase (Van Deun et al. 2017) under the following accession numbers: EV250092 for FBS‐derived EVs and EV250104 for milk‐derived EVs.

### 2.2 Isolation of FBS‐Derived EVs (FBS‐EVs)

**FIGURE 1:**
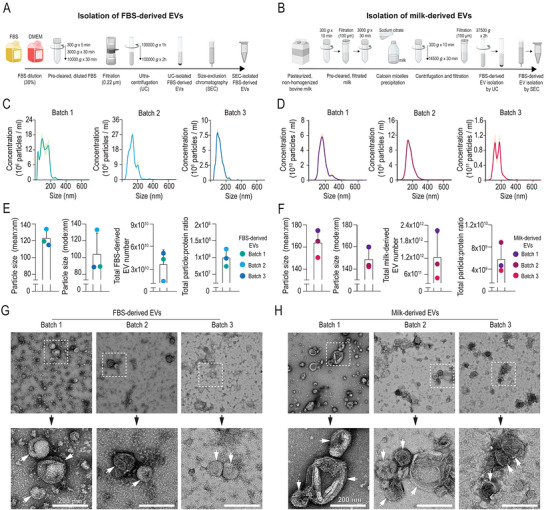
Isolation and characterisation of FBS‐ and milk‐derived EVs. **A**. Schematic overview of the FBS‐derived EV isolation workflow. Fetal bovine serum (FBS) was diluted with DMEM/F12 medium supplemented with GlutaMAX, followed by sequential centrifugation to remove debris. The supernatant was then filtered and subjected to ultracentrifugation. The resulting EV pellet was further purified by size‐exclusion chromatography (SEC). **B**. Schematic overview of the milk‐derived EV isolation workflow. Commercial pasteurised milk was processed through sequential centrifugation and filtration, followed by calcein micellae precipitation using sodium citrate (2%). The resulting suspension was centrifuged and filtered again, then ultracentrifuged. EVs were obtained from the final pellet by SEC. C–D. Nanoparticle tracking analysis (NTA) of FBS‐derived EVs (left) and milk‐derived EVs (right), showing particle concentration and size distribution. **E**–**F**. Quantitative analysis of NTA data for FBS‐derived EVs (left) and milk‐derived EVs (right), including mean and mode particle size, particle concentration, and particle‐to‐protein ratio. **G**–**H**. Representative transmission electron microscopy (TEM) images of FBS‐ and milk‐derived EVs.

Heat‐inactivated fetal bovine serum (FBS; Gibco, Cat. No. 10082147) was diluted to 30% (v/v) in DMEM/F‐12 medium supplemented with GlutaMAX (Gibco, Cat. No. 31331093), followed by sequential centrifugation steps to remove potential protein aggregates and larger vesicles (Figure 1A). The diluted FBS was first centrifuged at 300×g for 5 min at 4°C, then at 3,000×g for 30 min at 4°C, and subsequently at 10,000×g for 30 min at 4°C. The resulting supernatant was passed through 0.22 µm bottle‐top vacuum filters (Nalgene Rapid‐Flow, Cat. No. 568‐0020). Next, the filtered supernatant was aliquoted in ultra‐clear tubes (Beckman Coulter, catalogue number: 344059) and subjected to ultracentrifugation at 100,000×g for 1 h at 4°C using the Optima L‐90K ultracentrifuge (SW 40 Ti rotor). The EV‐containing pellet was gently resuspended in 0.22 µm‐filtered phosphate‐buffered saline (PBS, 1X), and a second ultracentrifugation step was performed under the same conditions for 2 h. The final pellet was resuspended in 500 µL of 0.22 µm‐filtered PBS (1X) and purified using size‐exclusion chromatography (SEC) as previously described (Marchan‐Alvarez et al. 2024). Briefly, the resuspended EVs were loaded onto Gen 2 SEC columns (70 nm pore size; Izon Science), and fractions 7 to 10, corresponding to the EV‐enriched eluate, were collected and pooled (∼2 mL) according to the manufacturer's protocol. The pooled fractions were concentrated to ∼100 µL using Vivaspin 2 centrifugal filters with a 10 kDa molecular weight cut‐off (Merck, Cat. No. Z614238). Final EV preparations were stored in 0.22 µm‐filtered PBS (1X) using low‐retention polypropylene microcentrifuge tubes (Axygen Maxymum Recovery, Corning, Cat. No. MCT‐150‐L‐C). Three individual preparations of FBS‐EVs, referred to as “batches”, were prepared for analysis.

### 2.3 Isolation of Milk‐Derived EVs (milk‐EVs)

EVs were isolated from undiluted, pasteurized (72°C for 15s), non‐homogenized bovine milk (Arla lantmjölk, 500 mL per isolation) purchased on the day of processing (Figure 1B). After gentle homogenization by inversion, milk was centrifuged at 300×g for 10 min at 4°C to remove cellular debris, and the resulting cream layer was carefully displaced to allow collection of the underlying milk. The supernatant was filtered through a 100 µm cell strainer (Falcon. Cat. No. 352360) and subjected to a second centrifugation at 3,000×g for 30 min at 4°C, followed by cream layer removal as described above. The clarified milk was then mixed 1:1 (v/v) with a freshly prepared 4 % sodium citrate solution (Merck, Cat. No. 1.06448) to reach a final concentration of 2 %, and incubated on a gentle rocker at 4°C for 15 min. After repeating the 300×g and 3,000×g centrifugation steps, the supernatant was transferred into ultracentrifuge tubes (Beckman Coulter. Cat. No. 355622) and ultracentrifuged at 16,500×g for 30 min at 4°C using a Beckman Coulter ultracentrifuge (Type 45 Ti rotor). The supernatant was further ultracentrifuged at 100,000×g for 2 h at 4°C to pellet EVs. The resulting pellets were resuspended in PBS (1X) using gentle pipetting and strained through a 100 µm cell strainer. Two additional washing steps were performed in PBS with intermittent straining. Final EV suspensions were prepared in 15–30 mL of PBS, filtered again using a 100 µm cell strainer, and rotated overnight at 4°C to ensure complete dispersion. EV preparations were stored at −80°C until further analysis.

Following ultracentrifugation‐based isolation, at least 3 mL of milk‐derived EVs or a complete EV batch deriving from 80–160 mL of milk was subjected to further processing. To minimize filter clogging by large aggregates, samples were first centrifuged at 300×g for 2 min at 4°C. The milk‐derived EV preparations were sequentially filtered through 0.8 µm and 0.22 µm sterile filters, pre‐wetted with PBS. For size‐based purification, samples were applied to qEVoriginal size‐exclusion chromatography columns (70 nm; Izon Science). EV‐enriched fractions (fractions 7–10) were collected in 2 mL microcentrifuge tubes. To concentrate the EV‐containing fractions, 30 kDa molecular weight cutoff centrifugal filters (Amicon. Cat. No. UFC9030) were preconditioned by spinning PBS (1X) at 3,000×g until the membrane was equilibrated. SEC fractions were then loaded and centrifuged at 3,000×g for 8 min at 4°C. Multiple spins were typically required to reduce the volume to 700–900 µL for mEVs, while flow‐through fractions concentrated more rapidly. The EVs were recovered by pipetting 100 µL of PBS along the filter membrane to elute residual vesicles adhered to the surface. The final concentrated EVs were transferred to cryovials (Sarstedt. Cat. No. 72.377 or 72.379) and stored at ‐80°C for downstream applications. Three individual preparations of milk‐EVs, referred to as “batches”, were prepared for analysis.

### 2.4 Nanoparticle Tracking Analysis (NTA)

Particle concentration and size distribution of each EV preparation were assessed using NTA as described previously (Marchan‐Alvarez et al., 2024). Samples were diluted in 0.22 µm‐filtered PBS (1X) and analysed using a NanoSight LM10‐HS instrument (Malvern Panalytical Ltd) equipped with a 488 nm laser and NTA 3.0 software. For each sample, five 30‐second videos were captured in light scatter mode under consistent camera settings (level 13), using a syringe pump at a flow rate of 50, all at room temperature. Analysis parameters were kept constant across all measurements, including a screen gain of 10 and a detection threshold of 3.

### 2.5 Protein Quantification in FBS‐ and Milk‐Derived EV Preparations

Protein concentration for the FBS‐derived EVs was quantified using the Micro BCA Protein Assay Kit (Thermo Scientific. Cat. No. 23235) as previously reported (Marchan‐Alvarez et al., 2024). In brief, 5 µL of EV sample was mixed with 145 µL of the Micro BCA working reagent. Next, a standard curve was generated using serial dilutions of the albumin standard supplied by the manufacturer to determine protein concentrations. Absorbance was measured at 562 nm using a spectrophotometer (BMG Labtech, FLUOstar omega). The particle‐to‐protein ratio was then calculated by dividing the EV particle count obtained via NTA by the corresponding protein concentration measured from freshly isolated EVs. The protein concentration of the milk‐derived EV preparations was assessed using DC protein assay (BioRad. Cat. No. 5000112) following the manufacturer's recommendations. Briefly, 20 µL of (diluted) EV sample or protein standard was mixed with 10 µL of reagent A, after which 80 µL of reagent B was added. The plate was read at 650 nm.

### 2.6 Transmission Electron Microscopy (TEM) of FBS‐ and Milk‐Derived EVs

EVs were prepared for TEM using negative staining as we previously reported (Marchan‐Alvarez et al., 2024). Briefly, 5 µL of each EV sample was applied to a glow‐discharged, carbon‐coated grid and incubated for 1 min. Excess liquid was gently blotted with filter paper, followed by a 7‐second incubation with 5 µL of 1 % uranyl acetate in water for contrast enhancement. After removing the excess stain with filter paper, the grids were allowed to air dry. Imaging was performed using a Talos 120C G2 transmission electron microscope (Thermo Scientific) operating at 120 kV and equipped with a CETA‐D camera.

### 2.7 Liquid Chromatography Electrospray Ionization Tandem Mass Spectrometry (LC‐ESI‐MS/MS)

Online LC‐MS analysis was conducted using a Dionex UltiMate 3000 RSLCnano System coupled to an Orbitrap Exploris 480 mass spectrometer (Thermo Scientific). An aliquot of approximately 2 µg of each sample was suspended in LC mobile phase A. Equal peptide amounts derived from each EV sample (FBS‐ and milk‐derived) were injected into the LC–MS/MS system to minimize technical variability; this approach ensures that differences observed across samples reflect biological variation rather than disparities in sample concentration. Peptides were first trapped on a C18 guard desalting column (Acclaim PepMap 100, 75 µm × 2 cm, nanoViper, C18, 5 µm, 100 Å) and subsequently separated on a 25 cm C18 column (Aurora Ultimate, C18, 1.7 µm, 25 cm × 75 µm). The mobile phase (nano capillary) consisted of solvent A (0.1 % formic acid in water) and solvent B (0.1 % formic acid in acetonitrile). Using a constant flow rate of 0.25 µL min^−1^, a gradient was applied, starting at 6 % B and increasing to 43 % B over 180 min, followed by a sharp rise to 100 % B over 5 min.

Data acquisition was performed with FTMS master scans at a resolution of 120,000 (400–1200 m/z). Data‐dependent MS/MS (60,000 resolutions) was performed on the top five most abundant ions using higher energy collision dissociation (HCD) with a normalized collision energy of 30 %. Precursor ions were isolated with a 2 m/z window. Automatic gain control (AGC) targets were set to 1 × 10^6^ for MS1 and 2 × 10^5^ for MS2, with maximum injection times of 100 ms for both MS1 and MS2. The total duty cycle duration was approximately 2.5 s. Dynamic exclusion was employed with a duration of 45 s, and precursors with unassigned or charge state 1 were excluded. An underfill ratio of 1 % was applied.

### 2.8 Peptide and Protein Identification

Orbitrap raw MS/MS files were converted to mzML format using msConvert from the ProteoWizard tool suite. Spectral data were analyzed using MSGF+ (v2020.03.14) (Kim and Pevzner 2014) and Percolator (v3.5) (Granholm et al. 2014) for target‐decoy Percolator scoring. All searches were performed against the bovine protein database Ensembl 104 within a proteomics workflow (https://github.com/lehtiolab/ddamsproteomics, v2.18), which was constructed using the Nextflow workflow tool (v22.10.5). MSGF+ search parameters included a precursor mass tolerance of 10 ppm, fully tryptic peptides, a maximum peptide length of 50 amino acids, and a maximum charge state of 6. Carbamidomethylation of cysteine residues was set as a fixed modification, while oxidation of methionine residues was considered as a variable modification. MS1 feature detection and quantification were conducted using Hardklor (v2.3.0) and Kronik (v2.20). Peptide spectrum matches (PSMs) identified at a 1 % false discovery rate (FDR) were used to assign gene identities. Protein quantification was based on the average intensity of the top three highest‐intensity peptides, with peptide MS1 quantification defined by the ‘Best Intensity’, representing the intensity value at the apex of the feature elution profile. Note that proteins were included in the proteomics dataset based on this top‐three peptide quantification approach, without applying an explicit minimum peptide threshold.

Protein FDRs were calculated using the picked‐FDR method, with gene symbols as protein groups, and restricted to 1 % FDR (Savitski et al. 2015). For peptide identification, a precursor ion mass tolerance of 10 ppm and product ion mass tolerances of 0.02 Da for HCD‐FTMS. The search algorithm accounted for tryptic peptides with a maximum of two missed cleavages, with carbamidomethylation (C) as a fixed modification and oxidation (M) and deamidation (N) as variable modifications.

### 2.9 Missing Data Handling Approach

**FIGURE 2:**
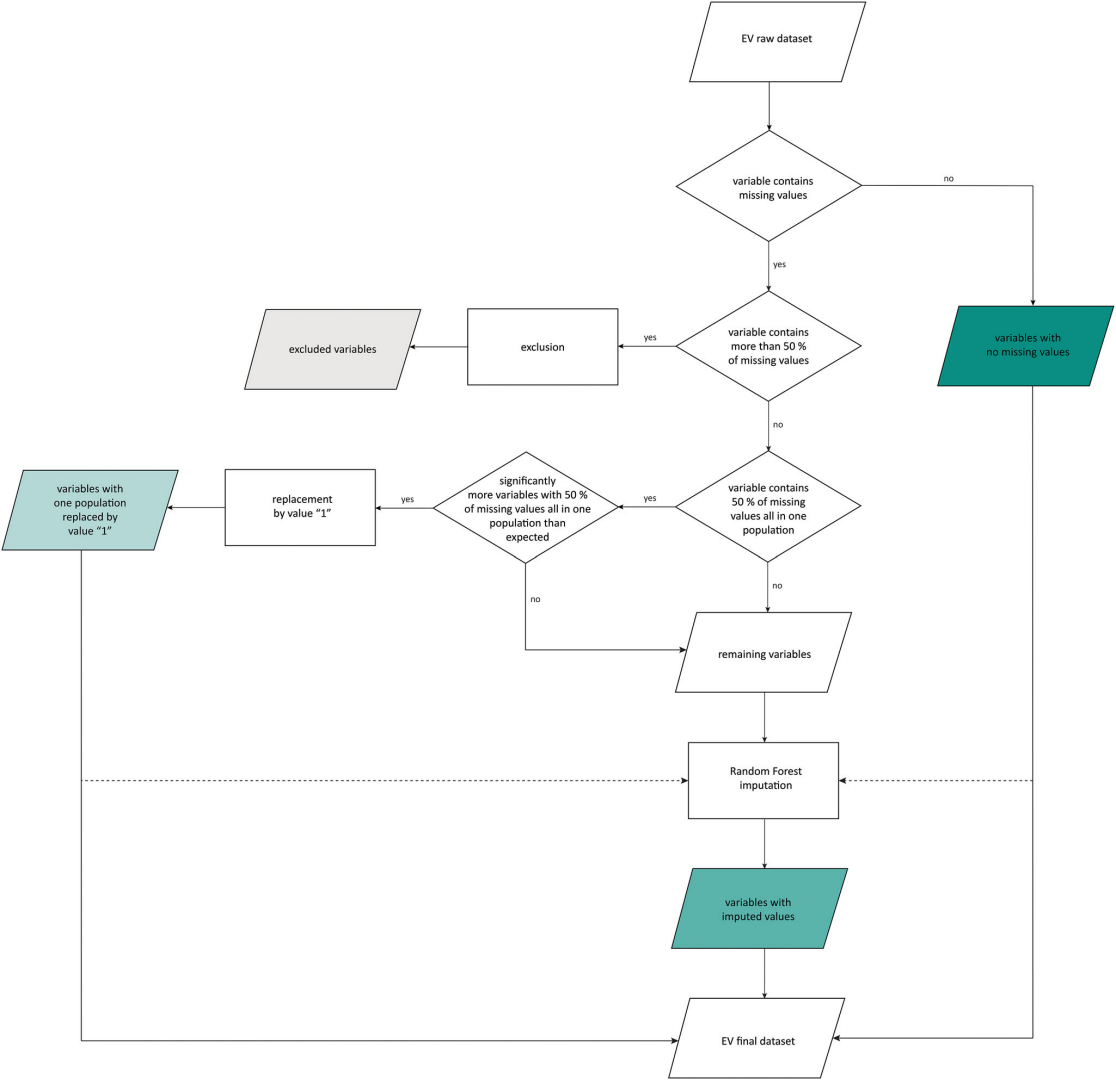
BioProEV workflow. From the EV raw dataset to the EV final dataset, different categorisations are applied to the variables (i.e. given proteins). According to the number of missing values, the variables are categorised as per the coloured boxes. The dashed arrows indicate that data represented by the associated boxes are included in the dataset used for the Random Forest (RF) imputation, by providing the remaining “forest” of values, but do not themselves undergo imputation by RF. The EV final dataset consists of all data from the green boxes pooled together.

We designed three conditions to handle the missing values (Figure 2):

1) To reduce the risk of overfitting, we excluded any variable (i.e. a given protein) in which more than half of the values were missing.
2) When all values in a variable were missing in one population, it is possible they are not missing randomly but due to protein absence/low expression in one of the EV populations; when this type of MNAR missing values (i.e. the type in which one population is different from another) was significantly enriched in the dataset, we considered this as biologically meaningful, and imputed the lowest possible value of “1” to replace these values in order to retain the biological differences.
3) In all other cases, missing values were handled using a Random Forest (RF) imputation algorithm.

We devised these three filtering and handling approaches with the following rationale. When most of the values for the same variable are missing, it would be difficult to impute with confidence using any strategy, since there are so few datapoints that can be used as a reference. To avoid biased imputation for those variables with a large number of missing values, we designed the first condition to filter them out (Figure 2, “excluded variables”). Here, we worked with six samples in total, so we removed any variables with at least 4 missing values from the data matrix, that is, more than 50 % of missing data for each variable.

For the second condition, we assumed that when one population, that is, FBS‐EVs or milk‐EVs, contained no missing values, and the other population contained only missing values, the missing values likely represent low value dropouts, that is, MNAR missing values (protein abundance‐dependence) (Jin et al., 2021). This assumption is based on a previous suggestion (Jin et al., 2021) that a variable completely missing in one population is more likely to be MNAR type. We assumed that these values have low abundance, which would mean that in the hypothetical situation where no values were missing and all values were plotted along an x‐axis based on abundance, these values would fall on the left side of the plot (i.e. have low scores); for this reason, they have been previously referred to as left‐censored missing values (Gardner and Freitas 2021). We reasoned that it is important not to exclude these variables as they likely represent proteins exclusively expressed in one population and therefore are highly meaningful. In this case, all missing values, represented by the placeholder “NaN” (not a number), were replaced by the lowest possible count value: “1” (Figure 2, “variables with one population replaced by value “1””). For example, a given variable, where FBS‐EVs = (NaN, NaN, NaN) and milk‐EVs = (12, 9, 14) would become FBS‐EVs = (1, 1, 1) and milk‐EVs = (12, 9, 14). However, importantly, three missing values in one population could also occur randomly (as MAR or MCAR) if for example, the two populations were biologically indistinguishable, protein abundance was generally low or there were analytical issues during sample processing. For this reason, we introduced a statistical test where this replacement by “1” was only implemented if there were significantly more variables containing three missing values in one population than expected by chance.

Finally, we used the RF method to impute the missing values in the remaining variables, that is, those containing one, two or three missing values distributed between both populations, and three missing values in one population if the number of these variables is not significantly different from their predicted occurrence by chance (Figure 2, “variables with imputed values”). We assumed that these missing values are of the MAR or MCAR type (protein abundance‐independence). The RF method is an iterative, supervised machine learning algorithm that estimates the final imputed values after a sequence of improving predictions (Breiman 2001; Stekhoven and Bühlmann 2012). It is suitable for proteomics data as it has high accuracy in terms of protein abundance and does not require understanding of the missingness mechanisms (Jin et al., 2021). First, it imputes all missing values using the mean calculation. Next, it fits a random forest on the observed data to predict the missing data for each variable with missing values. This process between observed and missing data continues until a stopping criterion or a maximum of iterations is reached, as previously described in detail (Stekhoven and Bühlmann 2012). The variables that had already passed conditions 1 and 2, that is, those originally contained no missing values, and those in which the missing values in one population replaced with value “1” were used for RF imputation. After imputation, the variables and their values passing these three conditions were combined into the “final dataset” (Figure 2, “EV final dataset”). The raw, filtered and final datasets can be found in the supplementary material “S1‐fbs‐milk‐evs‐datasets”.

To run these handling conditions sequentially, we combined them into a pipeline called BioProEV (***Bio*** logically‐relevant imputation of missing values in ***Pro*** teomic analyses of ***E*** xtracellular ***V*** esicles) (Miranda et al. 2025). It uses the missForest algorithm (Stekhoven and Bühlmann 2012), which is based on RF imputation technique, and has shown high accuracy handling missing data (Hong and Lynn 2020; Jin et al., 2021; Tang and Ishwaran 2017; Taylor et al. 2022). We included the missForest algorithm in the BioProEV using a Python implementation called MissForest (Yuen 2024), which uses RF features from scikit‐learn Python project (Pedregosa et al. 2011, Pedregosa et al. 2024) and has been applied in different types of data (Sacks et al. 2025; Shyam‐Sundar et al. 2024). The BioProEV bioinformatics pipeline is implemented in Python language and is freely available at https://doi.org/10.5281/zenodo.15440849, including further details in the “Readme” file.

Please note that this bioinformatics pipeline applies a default thresholding value of 50 % for step 1, that is, any variable in which more than half of the values are missing will be excluded. For purposes of exploration (outlined below in the results section), BioProEV includes an option for the user to adjust this value. The pipeline was developed to analyse the six samples using a threshold of more than 50 % of missing values for exclusion, that is, 4 and 5 NaN. However, without modification, it is also able to handle a different number of samples (e.g. 8, 10); however, it is limited to two populations with the same number of samples and considers only the amount of missing values, not the NaN configuration across samples, e.g. FBS‐EVs = (NaN, NaN, NaN) and milk‐EVs = (3, 2, NaN) is the same as FBS‐EVs = (NaN, 2, NaN) and milk‐EVs = (3, NaN, NaN), that is, four missing values in both cases. This means that the pipeline cannot differentiate between NaN configurations, restricting the inclusion/exclusion of specific configurations. Additionally, note that BioProEV was developed to classify MAR/MCAR and MNAR missing values based on patterns in the data, without distinction of sample type, allowing its application to EV datasets from any origin, e.g. serum, milk, blood, urine.

BioProEV was applied to the normalized data based on protein median, where the median protein ratio for each sample is 1. This ensures comparability across samples while preserving relative protein differences. Since imputation depends on the data structure, normalization was performed prior to imputation to avoid the artificial introduction of values that might distort the sample distribution (Karpievitch et al. 2012). Following normalization and replacement/imputation steps, the fold changes between the populations were calculated using the “EV final dataset”, and the resulting fold changes were log_2_‐transformed for downstream analysis.

### 2.10 Pathway Enrichment Analysis Using Reactome

We performed pathway enrichment analysis using Reactome, an open‐source, manually curated, and peer‐reviewed pathway knowledgebase and analysis platform (https://reactome.org/) (Fabregat et al. 2017; Griss et al. 2020; Milacic et al. 2024). Top 100 ranked genes from FBS‐ and milk‐derived EVs identify using LC mass spectrometry were used as input. Analyses were performed with default settings (including species set to Homo sapiens, interactors excluded) and pathways with a *p*‐value ≤ 0.05 were considered significantly enriched. The output included a ranked list of enriched pathways with summary statistics such as found entities and total entities. Voronoi visualization and detailed pathway inspection were conducted using the Reactome Pathway Browser.

### 2.11 Graphical Presentation

The data were presented in pie charts and volcano plots using Python (version 3.12). Venn diagrams were first generated in FunRich (http://www.funrich.org/) and presented here using Python (version 3.12). Violin plots were made using GraphPad Prism version 10.1.2 (324). Fisher´s exact test was performed using GraphPad Prism version 10.1.2 (324). To calculate the expected number of proteins within ExoCarta database, the total number of proteins listed in ExoCarta (8877) and the total number of proteins listed in Ensembl 104 database (excluding alternate isoforms) used for protein identification (21880) were used. FBS‐EV markers were selected from (X. Zhang et al. 2022) (“List of proteins detected in mouse blood EVs” supplementary), considering only those proteins with “Serum (average)” greater than 0. The selected data were filtered excluding 19 proteins with no gene names (verified in UniProt database [https://www.uniprot.org/]) and 3 duplicated proteins. Milk‐EV markers were selected from 38 individual studies gathered in (van Herwijnen et al. 2016). Normality of the data was assessed using the Shapiro‐Wilk test for plots in Figure 4B–D. The assumption of normality was met in all cases, and a statistical analysis was conducted using unpaired *t*‐test to evaluate differences between experimental groups, with significance levels denoted as p < 0.05 considered significantly different. 307 proteins were selected with “1 NaN: 3 NaN” configuration; however, only 160 proteins showed non‐zero variance within populations, allowing them for the statistical analysis. Note that zero variance in both populations for a given protein prevents its assessment of statistical significance. Gene ontology (GO) annotation plots were made with ShinyGO 0.82 (https://bioinformatics.sdstate.edu/go/). Schematics in Figure 1 were made with Biorender.com (agreement number: YH28KPW1Z2) and Reactome Pathway Browser. All final figures were made in Adobe Illustrator (version 29.4. Adobe Creative Cloud). A colour‐blind friendly palette was used in all figures.

## 3 Results

Three batches of EVs were isolated from FBS and bovine milk respectively, using source‐specific isolation methods coupled with SEC (Figure 1A,B). Characterization using NTA revealed that the isolated particles were smaller than 200 nm in diameter, and when coupled to the protein quantification assays, they had reproducible protein: particle ratios (Figure 1C–F). Isolated particles had the expected cup‐shaped (collapsed spherical) ultrastructures of EVs visualized by TEM (Figure 1G,H). Proteomic analysis of the six EV samples, with three biological replicates in each of two populations (FBS‐EVs and milk‐EVs), was performed by LC‐ESI‐MS/MS. Within the raw dataset, greater than half (85.1 %) of the variables (i.e. given proteins) contained at least one missing value. To handle missing values we devised a three step strategy: (i) we excluded any variable (i.e. a given protein) in which more than half of the values were missing; (ii) if a significantly greater number of variables that only contained missing values in one population were present than would be expected by chance, missing values were replaced with the lowest possible value of “1”; (iii) in all other cases, missing values were handled using a Random Forest (RF) imputation algorithm (Figure 2). The rationale behind this strategy is outlined in detail in the “Missing data handling approach” section, 2.9 of the Methods. We combined these steps into a bioinformatics pipeline called BioProEV.

**FIGURE 3:**
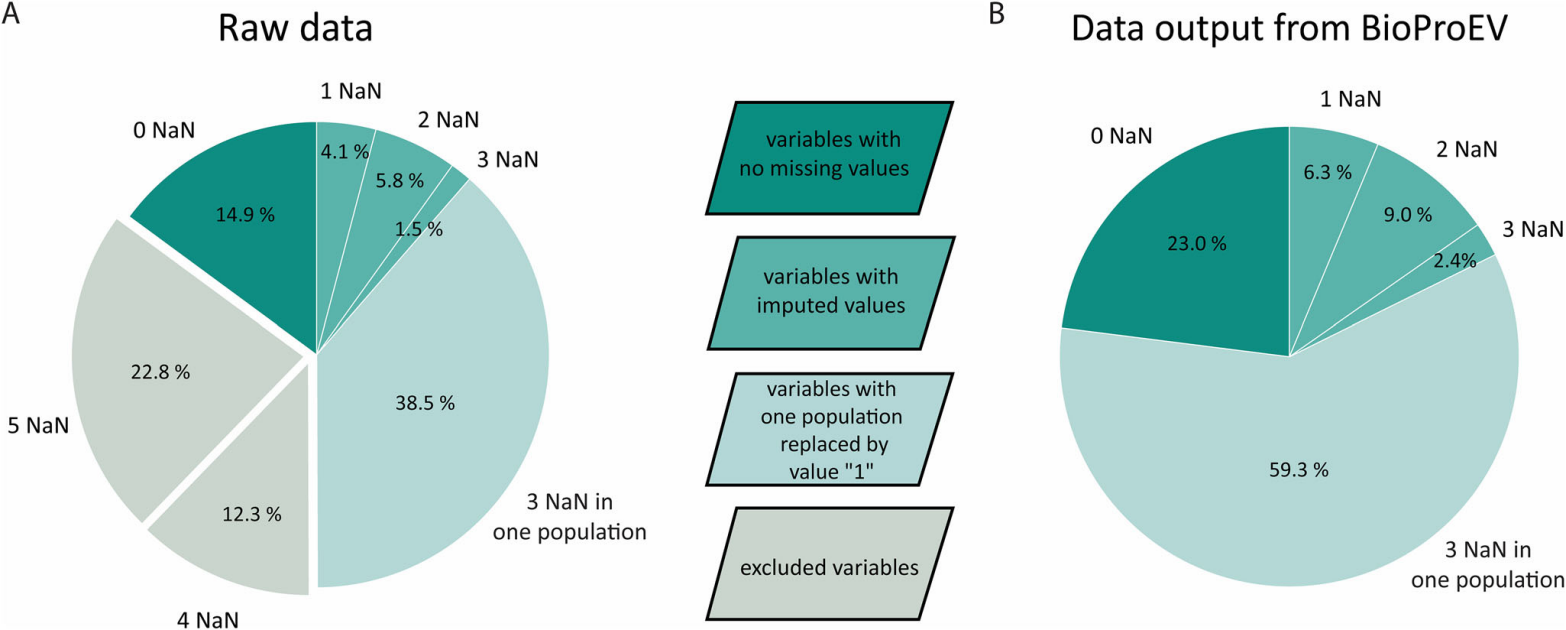
Distribution of non‐missing and missing data before and after using BioProEV. **A**. Analysis of missing value distribution among variables in raw dataset, showing the variables with observed data, that is, non‐missing data (0 NaN), one missing value (1 NaN), two missing values (2 NaN), three missing values distributed in both EV populations (3 NaN), three missing values exclusively in either FBS‐ or milk‐EVs (3 NaN in one population) and those variables with more than half of their values missing (4 NaN and 5 NaN). **B**. The variables with 4 and 5 missing values were excluded from the raw dataset, following the handling by BioProEV, and were therefore excluded in the data output and downstream analyses.

Based on the first criterion of BioProEV, 35.1 % of all variables were excluded from the dataset because more than half of the values across the six samples were missing (Figure 3A). All other variables present, the majority of which contained missing values (Figure 3B), were further processed before their eventual inclusion in the final dataset.

BioProEV applies two mechanisms to handle the missing data, depending on where the missing values are located. Three missing values in one population could represent MNAR values due to biological differences in protein expression; if these missing values were MAR or MCAR, then they would have an equal probability to appear in any of the six biological samples (Hong and Lynn 2020). To compare this with the raw dataset, we determined there would be 20 possible configurations (Table S1), where two of those configurations would reflect a situation when all missing values were in one population, giving a probability of 10 % (i.e. 2/20). However, in our raw dataset more than 95 % of the variables containing three missing values were configured in one population, which was significantly different from expected if they had been distributed at random (p <0,0001, using Fisher´s exact test). This analysis indicated that the missing values within the variables containing three missing values in a single population were not missing randomly; we therefore assumed that they were all MNAR values caused by very low protein expression in the sample, and therefore replaced these values with the lowest value in the dataset (i.e. “1”) to allow for subsequent quantification (Dabke et al. 2021). Please note that in cases where this assumption would not be met, based on the Fisher´s exact test, BioProEV will carry over these missing values and impute them with all remaining missing values using the RF algorithm (Figure 2).

**FIGURE 4:**
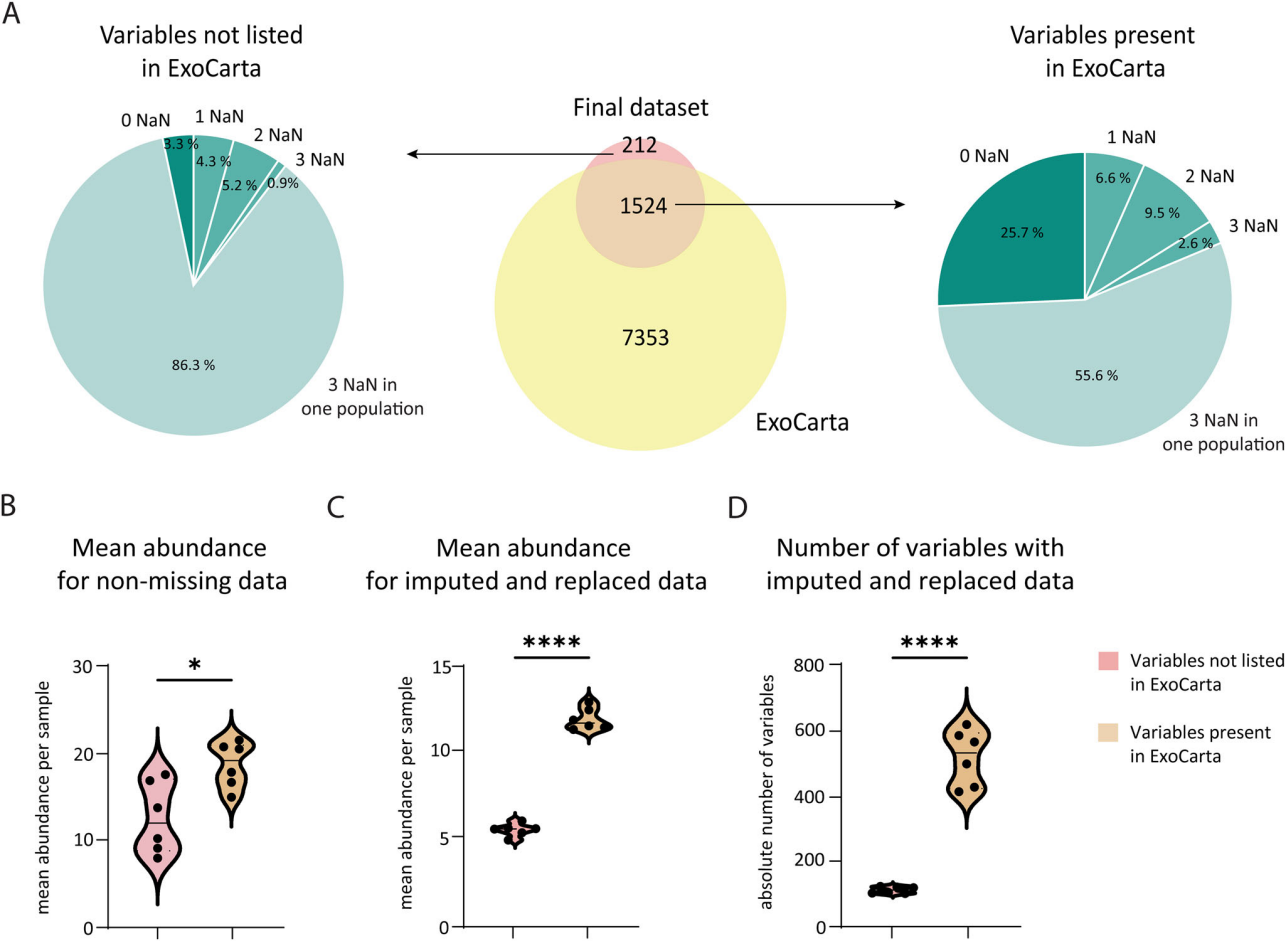
Data distribution in ExoCarta database. ExoCarta database was used to classify variables in the final dataset, which were analyzed by their original non‐missing and missing data rates according to the raw dataset. **A**. Pie charts presenting the distribution of non‐missing and missing data for variables classified by their presence in ExoCarta. **B**. Mean protein abundance per sample for variables with only non‐missing data; means were statistically compared using unpaired students *t*‐test (*p*‐value < 0.05). **C**. Mean abundance per sample for variables with imputed and replaced data; means were statistically compared using unpaired students *t*‐test (no significant difference). **D**. The absolute number of variables with imputed and replaced data; means were statistically compared using unpaired students *t*‐test (*p*‐value < 0.0001).

By applying BioProEV to this dataset, we could add 1336 variables to the 400 variables that contained no missing values, which generated a final dataset of 1736 variables. We next explored the composition of proteins sorted by their presence or absence from the ExoCarta database of small EV markers (Keerthikumar et al. 2016; Mathivanan et al. 2012; Mathivanan and Simpson 2009; Simpson et al. 2012). In the final dataset, 87.8 % of proteins were listed in the ExoCarta, indicating that the nanoparticles collected and isolated from FBS‐ and milk‐EVs samples were enriched with expected EV proteins (Figure 4A). Within the final dataset, 25.7 % of the proteins listed in ExoCarta originally contained no missing values in the raw dataset (i.e. NaN = 0), but this number was only 3.3 % for proteins not listed in ExoCarta (Figure 4A). We reasoned that there were two possible reasons for this: either most proteins present in our samples were listed in ExoCarta or the proteins not listed in ExoCarta had a lower abundance and therefore had an increased chance of dropping below detection thresholds and generating MNAR values (Jin et al., 2021). Indeed, among the variables with no missing values, those listed in ExoCarta had a significantly higher abundance than those not listed in this database (Figure 4B). While this was also true for the proteins in which missing values were imputed by BioProEV (Figure 4C), importantly, the majority of proteins (84.7 %) that were included in the final dataset after imputation were listed in ExoCarta (Figure 4D). The number of variables containing imputed values that were listed in ExoCarta was greater than the value that would be expected by chance (84.7 % observed versus 44,4 % expected; p <0,0001, Fisher´s exact test). Together, these results suggest that BioProEV allowed the inclusion of EV‐relevant proteins for downstream analyses.

**FIGURE 5:**
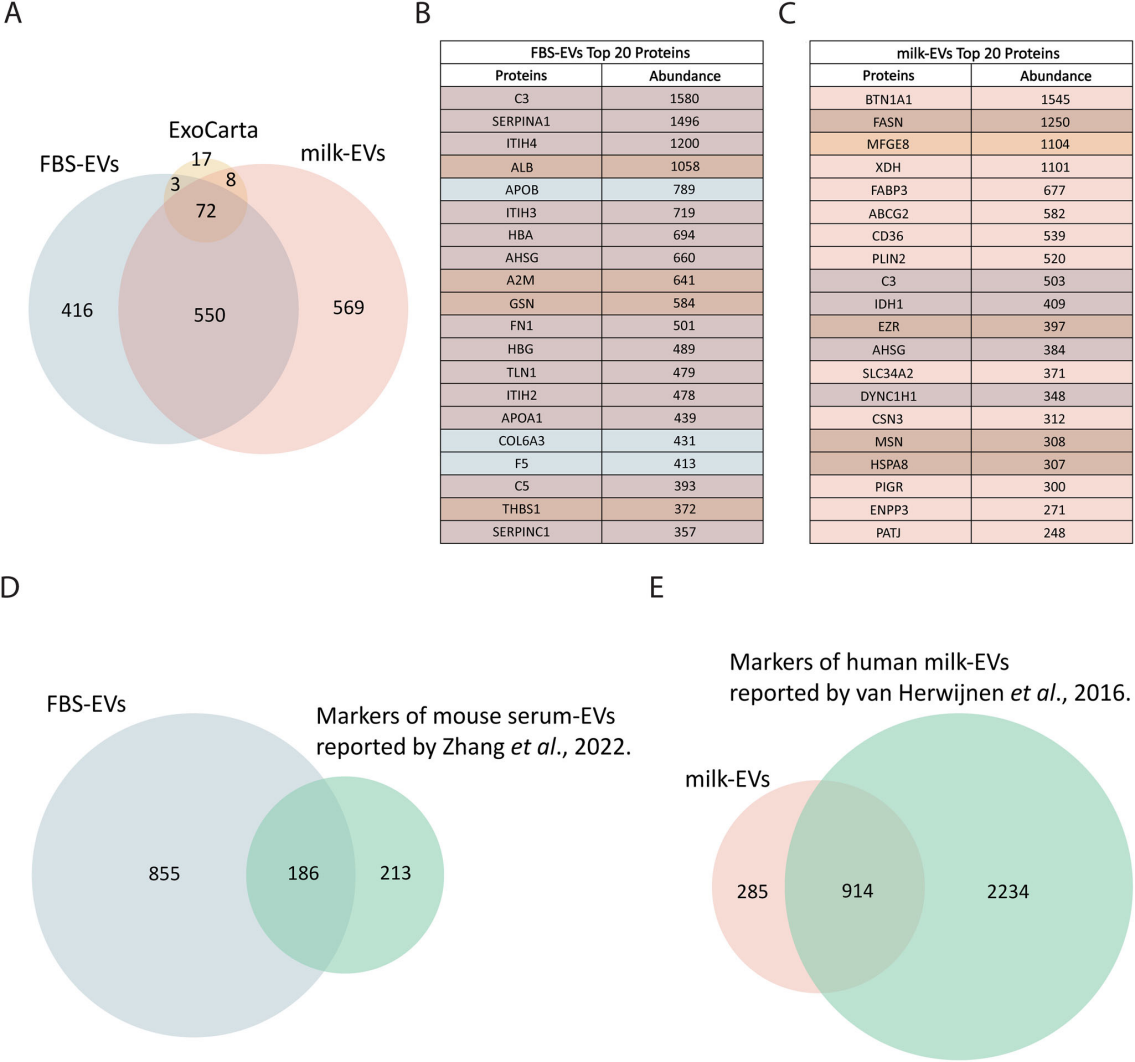
Most abundant proteins for FBS‐ and milk‐EVs in the final dataset. **A**. Venn diagram comparing the most abundant proteins identified in FBS‐ and milk‐EVs, with the 100 most frequently identified proteins in the ExoCarta database. **B** and **C**. The 20 most abundant proteins for both FBS‐ and milk‐EVs, respectively. Common proteins among the top 20 lists for both EV populations are highlighted using the color‐code used in A. **D** and **E**. Comparison of proteins identified in FBS‐ and milk‐EVs, with previous reported EV markers (van Herwijnen et al. 2016; Zhang et al. 2022). Please note that for analyses in this Figure, the total number of proteins in each population are not equal because when a protein contained three missing values in one population, it was considered absent and therefore removed from the list of identified proteins in that population.

To explore the proteins included in the two populations after missing value handling by BioProEV, we compared them to the 100 most frequently reported small EV markers, according to ExoCarta; a large proportion of proteins (≥ 75%) listed in ExoCarta were present in the FBS‐ and milk‐EVs populations (Figure 5A). Proteins associated with serum (e.g. APOB, F5, ITGA2B) and milk (BTN1A1, MFGE8, XDH) were among the most abundant proteins in each EV preparation (Figure 5B,C). Each population contained expected proteins, based on previous reports of similar EV preparations (van Herwijnen et al. 2016; X. Zhang et al. 2022). Almost half (46.6 %) of serum EV proteins previously described were found in our FBS‐EV list (Figure 5D), including the most abundant proteins such as C3, ITIH4 and ALB (Figure 5B). Additionally, more than three quarters (76.2 %) of the milk‐EV proteins we identified were reported in previous studies (van Herwijnen et al. 2016) (Figure 5E); the 18 most abundant milk‐EV proteins we identified were found within these markers (Figure 5C). These results indicate that in addition to maintaining EV‐relevant proteins in the dataset, the final dataset generated by BioProEV maintained source‐specific identifiers of EV populations.

**FIGURE 6:**
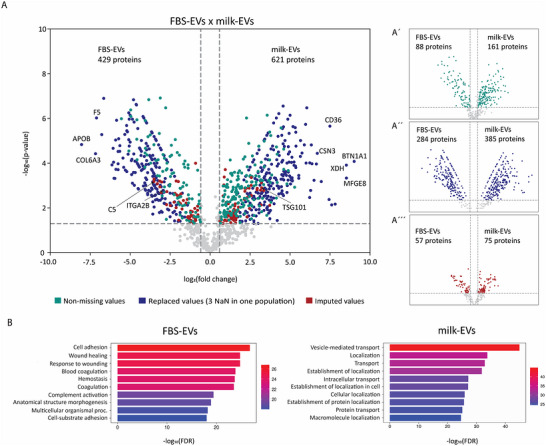
Differentially enriched proteins in FBS‐ and milk‐EV populations. **A**. Volcano plot showing the differential expression of proteins in the final dataset. The individual significantly different proteins (spots) are colour‐coded based on their handling classification: proteins without missing values (green), proteins with three missing values exclusively in one population (blue), and the proteins with remaining cases of missing values, which were imputed using RF method (red). Gray dots represent non‐significant proteins. A´‐A´´´ show each classification separately with respective non‐significant proteins. **B**. Top 10 biological process annotations for both FBS‐ and milk‐EVs. The plots were made with ShinyGO 0.82 using an FDR cutoff < 0.05.

**FIGURE 7:**
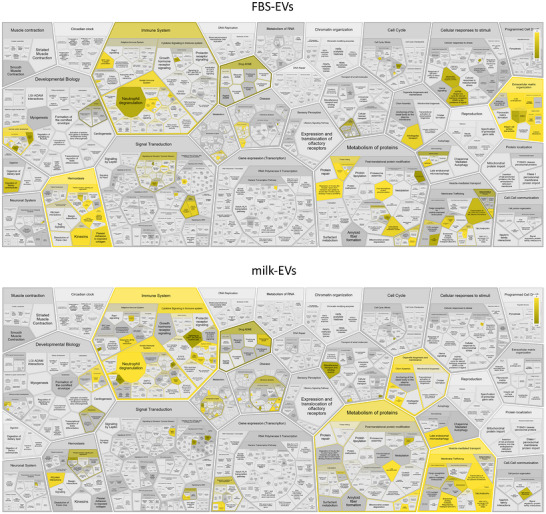
Pathway enrichment analysis for FBS‐ and milk‐derived EVs. **A**. Pathway enrichment analysis using Reactome for FBS‐ and milk‐derived EVs.

The final dataset was used to compare the proteomes of the EV populations. A considerable number of proteins were significantly enriched in either FBS‐ or milk‐EVs, 429 and 621 proteins, respectively. By specifically visualizing the replaced and imputed proteins, the impact of BioProEV became apparent (Figure 6A). Had all variables containing missing values been excluded from the dataset (as is typical in handling missing values by deletion (Han et al. 2023)), only 88 (FBS‐EVs) and 161 (milk‐EVs) significantly enriched proteins would have been identified (Figure 6A´). However, by using BioProEV, a large number of the proteins only containing values in one of the populations could be included (Fig. 6A´´). Examples include proteins for FBS‐EVs (e.g. APOB, ITGA2B and F5) and milk‐EVs (e.g. BTN1A1, XDH and MFGE8) preserved in the dataset. A further 57 (FBS‐EVs) and 75 (milk‐EVs) proteins handled via RF imputation were also significantly enriched between the populations (Fig. 6A´´´); these included proteins relevant to the samples, such as C5 and TSG101. Some proteins included by both approaches (replacement and imputation) were found in previously reported FBS‐ and milk‐EV markers, such as ITGA2B (X. Zhang et al. 2022) and CD36 (van Herwijnen et al. 2016). To test the relevance of the differentially enriched proteins, we conducted a gene ontology annotation analysis (Figure 6B; S1); biological processes associated with serum, such as cell adhesion and wound healing, were associated with proteins enriched in the FBS‐derived EV preparations, while vesicle‐mediated transport and localization were associated with milk‐EV preparations, indicating relevant proteins were identified in each population. These annotations were supported by a Reactome analysis of biological pathways (Figure 7).

To further test the validity of the handling approach, we questioned whether the cut‐off of 50% NaN used in step 1 might be too severe and exclude relevant proteins. To explore this possibility, we isolated the variables with four missing values in a configuration whereby three of the missing values were restricted to one population, e.g. FBS‐EVs = (NaN, NaN, NaN) and milk‐EVs = (3, 2, NaN), which we refer to as the “1 NaN: 3 NaN” configuration. We chose this configuration based on the prior reasoning that three missing values in one population indicated an absence of the protein (i.e. MNAR), and could be reasonably replaced by “1”; subsequently, the remaining missing value in each variable (MAR/MCAR based on our earlier reasoning) was handled by RF imputation (Fig. S2A). Analysis of the resulting data revealed that 61 proteins were significantly enriched between populations (Fig. S2B); with 32 and 80 of the proteins found in FBS‐ and milk‐EVs listed in ExoCarta, respectively (Fig. S2C). However, it should be noted that in datasets containing a greater proportion of missing values, or when stratification of populations/sub‐populations is uncertain, modifying this exclusion threshold could improve the biological relevance of the results. For such analyses or data exploration, simple adjustable thresholding is built in to step 1 of the BioProEV pipeline (detailed in section 2.9, “Missing data handling approach”).

Our findings demonstrate that missing value handling is of critical importance to the interpretation of EV proteomics data and that BioProEV, by identifying different types of missing values and handling them accordingly, can be used to maintain proteins of biological relevance in EV proteomics datasets.

## 4 Discussion

Traditional approaches to handling missing values normally include deletion and imputation (Han et al. 2023). The former is a simple case of removing all variables, that is, proteins in the case of proteomics data, with at least one missing value from the dataset. Deletion may introduce severe bias and loss of precision in the downstream analysis, leading to a loss of meaningful information (Little and Rubin, 2019; RUBIN 1976). By contrast, imputation replaces the missing data using either single or multiple imputation strategies, both of which have their pros and cons. The risk of single imputations is that the correlation between variables is ignored, which may lead to an underestimation of the data. For instance, a common single imputation is the mean calculation for each variable, which can be distorted by outliers and reduce the correlation between variables and the variance of the imputed values (Cox and Donnelly 2011; Little and Rubin, 2019). In the case of multiple imputations, the estimated value is predicted from the observed values in an iterative performance, similar to the previous description of RF algorithm. These imputation algorithms use a previous estimated data matrix to run a more accurate prediction (Little and Rubin, 2019). An important point here is that the multiple imputation procedure is able to keep most biologically meaning from data because it considers the relationship between data points. Several methods have been developed, such as RF algorithms (Stekhoven and Bühlmann 2012) and multivariate imputation by chained equations (MICE) (Buuren and Groothuis‐Oudshoorn 2011). These methods have shown a good performance for missing values from MCAR and MAR mechanisms (protein abundance‐independence), while MNAR missing values are complex to estimate, since they depend on the missing data (protein abundance‐dependence), leading to uncertainty in the results (J. Li et al. 2024). Understanding the missingness mechanism would be ideal to guide how to handle the missing data, however there is no standard rule or test to identify it. Although it is possible to test for MCAR, identifying MAR and MNAR are not testable (Little and Rubin, 2019; van Buuren 2018).

To retain biological information, we took advantage of the possibility to identify MNAR missing values by comparing observed data with expected data using statistical testing. By replacing these MNAR values with the lowest possible value “1” we could retain the biological relevance differences between populations. The advantage of this approach using the example dataset is clear from the color‐coded volcano plot in Figure 6, demonstrating the large number of differentially expressed proteins that would have been excluded by a deletion strategy (Hill et al. 2008; Oberg et al. 2008; Wang et al. 2003). While this approach may underestimate true fold changes of a protein between the populations (since the values of absent proteins are likely to be below the value of 1), it has the advantage of increasing the chance that a protein will appear as significantly different between populations during statistical testing (because the variance of the replaced values [i.e. 1, 1, 1] is 0), resulting in meaningful biological differences between the populations. Another advantage of this method was that RF imputation was used for MCAR/MAR but not MNAR missing values, which it can predict with less accuracy (J. Li et al. 2024). Therefore, the two imputation approaches were well suited to the types of missing values they were applied to. Hence, using BioProEV we were able to keep a considerable number of variables in the final dataset for the downstream analysis and maintain biological relevance allowing us to avoid loss of critical information. The imputation of small values is similar to the MinDet method; however, unlike MinDet, we classified missing values by variable, rather than independently, allowing us to better screen those most likely to be MNAR (Gardner and Freitas 2021).

To exemplify the logic of this strategy, if observed values are exclusively present in one population at either high, e.g. protein A: (population #1: 44, 42, 40), (population #2: NaN, NaN, NaN) or moderate levels e.g. protein B: (population #1: 11, 12, 11), (population #2: NaN, NaN, NaN), then all missing values will be replaced by the value “1” (if there are significantly more variables with 50 % of missing values all in one population than would be expected by chance). As the missing values are exclusively in one population (i.e. for both proteins A and B), our assumption is that the missing values are missing not at random (MNAR) and therefore, replacement by “1” will increase the chances of statistical differences between populations, enabling these proteins to be highlighted within the dataset. However, the fold change between protein levels in the populations will be greater for protein A than protein B (i.e. 42 versus 11), retaining the biological information within the observed data in the dataset based on protein abundance. On the other hand, in the theoretical case of protein C (population #1: 11, 8, NaN), (population #2: 10, NaN, NaN), there would be a greater chance that the missing values in the second population would be MAR/MCAR, than those in proteins A and B; therefore, to better retain fold‐change differences between populations present in the observed data (in this case 11 and 8 in population #1, and 10 in population #2) random forest imputation was used instead of replacement by the value “1”. Even though the number of variables with three missing values in one population occurred more frequently than by chance in the dataset, replacing all of these missing values by “1” introduces a small number of errors into the data. This is because among these variables, some will have three missing values which are MAR or MCAR, and replacing the missing values by “1” may underestimate their true abundance. However, please note that distinguishing MAR and MCAR from MNAR in proteomics data with complete certainty is not possible, and all imputation approaches introduce some discrepancies; we deem this is an acceptable risk in order to retain the true biological differences that this approach allows. To strength the reliability of BioProEV output, independent experimental validation, such as western blotting, enzyme‐linked immunosorbent assay (ELISA) or targeted mass spectrometry, could be performed to verify low protein abundance/absence of novel targets appearing exclusively in one EV population.

While there remains no consensus on whether imputation should be performed at the peptide or protein level, in our case, we performed imputation on the protein level; the reason for this choice was because the three top ranked peptide values are combined to generate the protein value, a missing value at protein level indicates multiple peptides with missing values within that protein. This further increases the chance that when a variable contains missing values exclusively in one population at the protein level, these are MNAR (Jin et al., 2021).

The FBS‐ and milk‐derived EV datasets investigated in this work were acquired using LC‐ESI‐MS/MS in data‐dependent acquisition (DDA) mode. This approach selects the most abundant peptides within a range of time to fragmentation and subsequent identification and quantification. This approach tends to fail to detect low‐abundance proteins, resulting in a high prevalence of missing values (Zhou et al. 2018). In this case, although missing values still reflect low abundance due to biological reasons, the absence of a protein in the quantified data might be due to a technical factor (MAR type), that is, the limit of detection, and not to a true absence (MNAR type). An alternative approach is the data‐independent acquisition (DIA) mode that typically exhibits more complex peptide coverage and lower missing values (Zou et al. 2026). Although DIA mode is increasingly used, less expensive DDA‐based techniques are still widely employed, and both approaches have contributed to proteomic discoveries, such as cancer biomarker identification and protein characterization (Kong et al. 2022; Zou et al. 2026). As previously mentioned, the true missingness type cannot be determined, and one population that is entirely absent is more likely to be of the MNAR type than MAR. Therefore, our pipeline design is consistent for DDA data and could, in principle, be extended to DIA data.

Regarding reproducibility, BioProEV relies on the random forest algorithm that contains stochastic elements during training; therefore, it will not always yield precisely the same imputed values when applied to the same dataset. Nevertheless, fold changes and statistical analyses are only likely to be negligibly impacted by these variations.

Pipelines using different types of imputation to differentially handle both MAR/MCAR and MNAR in the same proteomics dataset have previously been called hybrid approaches, among of the most commonly used being KNN‐TN (K‐nearest neighbours truncation) and MNAR/MAR MI SFI (Multiple Imputation Selection‐Filter‐Imputation)‐hybrid methods. KNN‐TN (Shah et al. 2017) assumes that the complete data, that is, no missing values, follows a truncated distribution and considers the detection limit as the truncation point to classify the missingness types. KNN‐TN identifies the missingness type by estimating the distribution for the values in each variable; based on this distribution, if missing values in a given variable are predicted to lie at or below the detection limit, they are considered MNAR values, otherwise they are considered to be MAR/MCAR values. Since the missing values are differentiated, all missing values are imputed, in each case variables with similar profiles (i.e. “nearest neighbors”) are used to guide imputation. The MNAR/MAR MI SFI‐hybrid approach (Gardner and Freitas 2021) is based on the imputeLCMD (Left‐Censored Missing Data) R package (Lazar 2014). It classifies MAR/MCAR and MNAR types by assuming that the mean values of variables follow a normal distribution and differences from this are caused by the presence of MNAR values. Then the classified MAR/MCAR values are imputed by KNN method Cover and Hart 1967) and MNAR values by QRILC (quantile regression imputation of left‐censored data) method (Lazar 2014), where missing values are imputed separately for each population. While these methods have their strengths, BioProEV has several notable advantages since it (i) excludes variables with large numbers of missing values, (ii) prioritizes the identification of MNAR values based on biological differences between populations, and (iii) uses a highly effective Random Forest machine learning algorithm during imputation. For these reasons we believe BioProEV is a robust pipeline that may be better suited to handle missing values in order to retain biological information within EV proteomics datasets.

## 5 Limitations

One reason why there is no standardized approach to missing value handling in proteomics data is that each experimental set‐up comes with its own set of assumptions, and a one‐size‐fits‐all strategy cannot be easily justified. Within BioProEV, the user can adjust sample size and the exclusion threshold for the percentage of missing values within a variable (so that variables with as many as 90 % missing values could be retained). Hence, while this means BioProEV can be applied to a variety of datasets, some caution should be observed; for example, in a dataset with two populations of 10 replicates, a threshold of 7/20 observed values (i.e. 65 % missing values) may be justified when 10 of those missing values are in one population and only three are in the other population (since one group contains entirely missing values, it could be biologically relevant to replace those values by “1”, with the remaining missing values imputed), but when the missing values are spread between the populations (e.g. 6 missing values in one population, and 7 in the other population), BioProEV will handle these missing values by random forest imputation alone, relying on the few observed data—hence, in this case, confidence in biologically meaningful imputation decreases and the risk of overfitting becomes greater, potentially leading to erroneous results. Furthermore, uncertainty increases if populations contain known or unknown sub‐populations. These complex and indeterminate permutations have made us cautious to devise a standard pipeline to handle all missing value configurations. Hence, we have only validated BioProEV up to 50 % missing values, and other researchers must apply their own justification to go beyond this threshold. Nevertheless, the logic and justifications we have devised in this manuscript can be applied to diverse scenarios, depending on the experimental design.

Handling missing values is not a straightforward process, and several aspects must be considered to design an appropriate approach. Here, we addressed the different mechanisms of missingness and how they can be either replaced or imputed, e.g. deletion or mean imputation. BioProEV steps were designed based on prior studies, which demonstrated that the RF method performs well for the MAR/MCAR type and at the protein level, as well as one entirely missing population may be of MNAR type (Jin et al., 2021; Taylor et al. 2022). Furthermore, while BioProEV is expected to be consistent for other datasets, its broader application to DIA datasets has not been evaluated here and could be investigated in future work.

One additional limitation of our approach is that BioProEV applies a statistical test in an attempt to identify MNAR missing value type and, if these missing values occur more frequently than by chance, they are replaced by “1”. However, MCAR/MAR can also appear in the same configuration, and this strategy may underestimate the true protein abundance in these variables, introducing bias in the downstream analysis; further development using binomial (Xiao et al. 2013) or detectability‐based left‐censoring decision (Karpievitch et al. 2009) may improve this thresholding. However, the identification of missingness mechanisms (MCAR/MAR and MNAR) is an existing challenge in handling missing values and the risk in BioProEV workflow is plausible to retain biologically meaningful information.

## 6 Conclusion

We highlight the importance of missing value handling to proteomic analysis of EVs, and present a pipeline that can be used to handle such missing values with a focus on retaining biologically‐relevant data EVs. We arranged this pipeline, named BioProEV, which contains a built‐in decision‐making algorithm for hybrid imputation that can be used to deal with EV samples from two biological populations.

## Author Contributions

**Pâmella Miranda**: Conceptualization, Data curation, Investigation, Formal analysis, Methodology, Project administration, Software, Visualization, Writing – original draft, Writing – review and editing. **Jose G. Marchan‐Alvarez**: Investigation, Methodology, Visualization, Writing – original draft, Writing – review and editing. **Annemarijn Offens**: Methodology, Writing – review and editing. **Ruihan Zhou**: Methodology, Writing – review and editing. **Mathieu Y. Brunet**: Methodology. **Loes Teeuwen**: Methodology, Writing – review and editing. **Maria Eldh**: Methodology, Writing – review and editing. **Susanne Gabrielsson**: Resources, Conceptualization, Supervision, Writing – review and editing. **Phillip T. Newton**: Conceptualization, Project administration, Resources, Funding acquisition, Supervision, Writing – original draft, Writing – review and editing.

## Conflicts of Interest

The authors have no conflicts of interest to declare.

## Supporting information




**Supplementary Figure S1**: jex270150‐sup‐0001‐FigureS1.tif

**Supplementary Figure S2**: jex270150‐sup‐0002‐FigureS2.tif

**Supplementary Table S1**: **Table S1**. All possible configurations of observed (Value) and missing value (NaN) across all six EV samples

**Supporting Information**: jex270150‐sup‐0004‐SuppMat.xlsx

**Supporting Material**: jex270150‐sup‐0005‐SuppMat.docx

## Data Availability

BioProEV pipeline can be found at https://doi.org/10.5281/zenodo.15440849, and the FBS‐ and milk‐EVs raw, filtered and final datasets used in this work in the supplementary material “S1‐fbs‐milk‐evs‐datasets”.
