# Supplementary figures and images for: BioProEV: A Bioinformatics Pipeline for Biologically‐Relevant Handling of Missing Values in the Analysis of Extracellular Vesicles by Mass Spectrometry
Source: J Extracell Biol. 2026 May 15;5(5):e70150. doi: 10.1002/jex2.70150 (PMC13178795; doi:10.1002/jex2.70150)

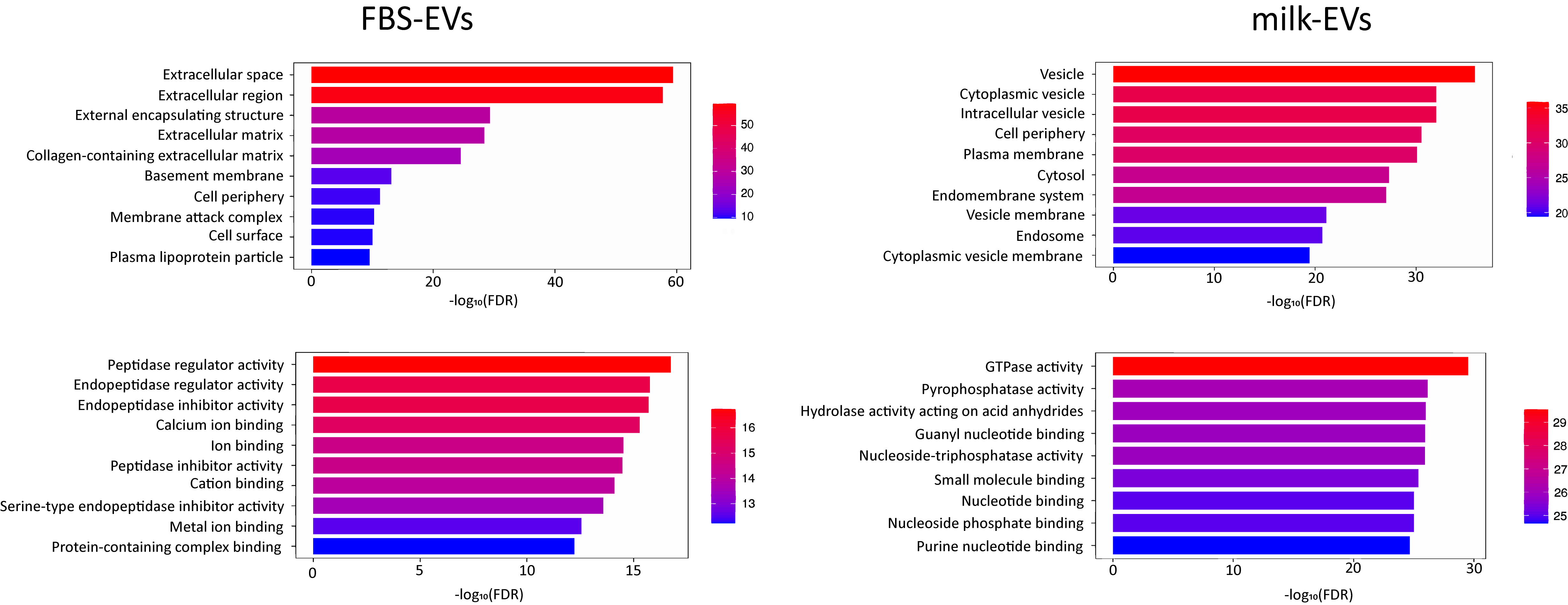

Supplement: Supplementary file 1 — Supplementary Figure S1: jex270150‐sup‐0001‐FigureS1.tif [file JEX2-5-e70150-s004.tif]

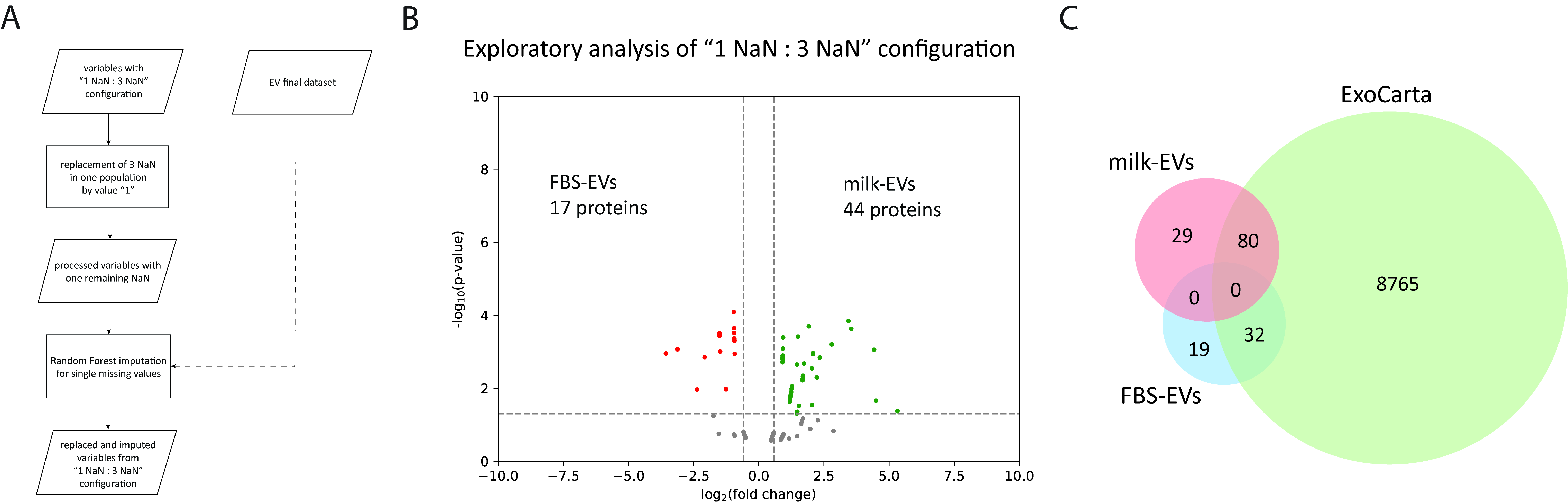

Supplement: Supplementary file 2 — Supplementary Figure S2: jex270150‐sup‐0002‐FigureS2.tif [file JEX2-5-e70150-s003.tif]
