# Supplementary material for: BioProEV: A Bioinformatics Pipeline for Biologically‐Relevant Handling of Missing Values in the Analysis of Extracellular Vesicles by Mass Spectrometry
Source: J Extracell Biol. 2026 May 15;5(5):e70150. doi: 10.1002/jex2.70150 (PMC13178795; doi:10.1002/jex2.70150)
Supplement: Supplementary file 3 — Supplementary Table S1: Table S1. All possible configurations of observed (Value) and missing value (NaN) across all six EV samples [file JEX2-5-e70150-s001.pdf]

Table S1. All possible configurations of observed (Value) and missing value (NaN) across all six EV samples.

		Biological samples					
		FBS-EVs			milk-EVs		
		**1**	**2**	**3**	**4**	**5**	**6**
**Configuration**	**1**	Value	Value	Value	NaN	NaN	NaN
	**2**	Value	Value	NaN	Value	NaN	NaN
	**3**	Value	Value	NaN	NaN	Value	NaN
	**4**	Value	Value	NaN	NaN	NaN	Value
	**5**	Value	NaN	NaN	Value	Value	NaN
	**6**	Value	NaN	NaN	Value	NaN	Value
	**7**	Value	NaN	NaN	NaN	Value	Value
	**8**	Value	NaN	Value	Value	NaN	NaN
	**9**	Value	NaN	Value	NaN	Value	NaN
	**10**	Value	NaN	Value	NaN	NaN	Value
	**11**	NaN	Value	Value	Value	NaN	NaN
	**12**	NaN	Value	Value	NaN	Value	NaN
	**13**	NaN	Value	Value	NaN	NaN	Value
	**14**	NaN	Value	NaN	Value	Value	NaN
	**15**	NaN	Value	NaN	Value	NaN	Value
	**16**	NaN	Value	NaN	NaN	Value	Value
	**17**	NaN	NaN	Value	Value	Value	NaN
	**18**	NaN	NaN	Value	Value	NaN	Value
	**19**	NaN	NaN	Value	NaN	Value	Value
	**20**	NaN	NaN	NaN	Value	Value	Value
