# Supplementary material for: BioProEV: A Bioinformatics Pipeline for Biologically‐Relevant Handling of Missing Values in the Analysis of Extracellular Vesicles by Mass Spectrometry
Source: J Extracell Biol. 2026 May 15;5(5):e70150. doi: 10.1002/jex2.70150 (PMC13178795; doi:10.1002/jex2.70150)
Supplement: Supplementary file 5 — Supporting Material: jex270150‐sup‐0005‐SuppMat.docx [file JEX2-5-e70150-s005.docx]

**Figure S1. Gene ontology (GO) annotation for FBS- and milk-derived EVs.**

Cellular component (above) and molecular function (below) annotations for FBS- and milk-EVs.

**Figure S2. Exploratory analysis of four missing values in “1 NaN : 3 NaN” configuration.**

**A.** Workflow applied to explore the handling of proteins with four missing values in the following configuration: a single missing value in one population and three missing values in the other (“1 NaN : 3 NaN”), e.g. FBS-EVs = (NaN, 3, 2) and milk-EVs = (NaN, NaN, NaN), or FBS-EVs = (NaN, NaN, NaN) and milk-EVs = (3, NaN, 2). The 3 NaN in one population were replaced by value “1”, then the single missing value in one population is imputed using RF imputation. As indicated, for this exploration, the EV final dataset was used as the reference data matrix for the imputation step. **B.** Differential expression of proteins with “1 NaN : 3 NaN” configuration. Red/green dots indicate those proteins significantly enriched in FBS- and milk-derived EVs, respectively. **C.** Venn diagram presenting identified proteins listed in the ExoCarta database. Please note that proteins were considered absent from a population when they contained three missing values in that population, and were therefore excluded from the Venn diagram.
